# A Bacteriochlorin‐Based Metal–Organic Framework Nanosheet Superoxide Radical Generator for Photoacoustic Imaging‐Guided Highly Efficient Photodynamic Therapy

**DOI:** 10.1002/advs.201900530

**Published:** 2019-05-16

**Authors:** Kai Zhang, Zhaofeng Yu, Xiangdan Meng, Weidong Zhao, Zhuojie Shi, Zhou Yang, Haifeng Dong, Xueji Zhang

**Affiliations:** ^1^ School of Materials Science and Engineering University of Science & Technology Beijing 30 Xueyuan Road Beijing 100083 P. R. China; ^2^ Tianjin Key Laboratory of Radiation Medicine and Molecular Nuclear Medicine Institute of Radiation Medicine Chinese Academy of Medical Science and Peking Union Medical College Tianjin 300192 P. R. China; ^3^ Beijing Key Laboratory for Bioengineering and Sensing Technology Research Center for Bioengineering and Sensing Technology School of Chemistry & Biological Engineering University of Science & Technology Beijing 30 Xueyuan Road Beijing 100083 P. R. China

**Keywords:** metal–organic frameworks, photoacoustic imaging, photodynamic therapy, photosensitizer anion radical, superoxide anion radical

## Abstract

Hypoxic tumor microenvironment is the bottleneck of the conventional photodynamic therapy (PDT) and significantly weakens the overall therapeutic efficiency. Herein, versatile metal–organic framework (MOF) nanosheets (DBBC‐UiO) comprised of bacteriochlorin ligand and Hf_6_(µ_3_‐O)_4_(µ_3_‐OH)_4_ clusters to address this tricky issue are designed. The resulting DBBC‐UiO enables numerous superoxide anion radical (O_2_^−•^) generation via a type I mechanism with a 750 nm NIR‐laser irradiation, part of which transforms to high toxic hydroxyl radical (OH•) and oxygen (O_2_) through superoxide dismutase (SOD)‐mediated catalytic reactions under severe hypoxic microenvironment (2% O_2_), and the partial recycled O_2_ enhances O_2_^−•^ generation. Owing to the synergistic radicals, it realizes advanced antitumor performance with 91% cell mortality against cancer cells in vitro, and highly efficient hypoxic solid tumor ablation in vivo. It also accomplishes photoacoustic imaging (PAI) for cancer diagnosis. This DBBC‐UiO, taking advantage of superb penetration depth of the 750 nm laser and distinct antihypoxia activities, offers new opportunities for PDT against clinically hypoxic cancer.

Hypoxia, a quite general feature for most solid tumors (partial oxygen pressure <5 mm Hg), is generally caused by the aggressive proliferation of cancer cells and tumor vasculature distortion.^1^, ^2^, ^3^ Photodynamic therapy (PDT) utilizes a photosensitizer (PS) excited by an appropriate light irradiation to generate reactive oxygen species (ROS); in most of cases, it involves a process that the ground triplet‐state molecular oxygen (^3^O_2_) is transformed to the reactive singlet oxygen (^1^O_2_) via the type II mechanism extremely dependent on the concentration of oxygen (O_2_).^4^, ^5^, ^6^, ^7^ Thus, the O_2_ shortage in solid tumors significantly limits the anticancer capability of PDT, especially in cases that need continuous treatment.^8^, ^9^ To address this issue, various innovative strategies have been developed, such as O_2_‐replenishing nanosystem to deliver O_2_ (e.g., hemoglobin and perfluorocarbon)^10^, ^11^, ^12^, ^13^, ^14^ or oxygen self‐supplement nanomaterials to generate O_2_ (e.g., MnO_2_, Pt, CaO_2_, and catalase)^15^, ^16^, ^17^, ^18^, ^19^ in the tumor microenvironment to elevate the tumor O_2_ concentration and enhance the PDT efficacy. However, most of these nanocomposite systems undergo tedious fabrication procedures and complex toxicity evaluation owing to the multiple components. Hence, it is highly desirable to develop nanoagents that directly reduce the requirement of O_2_ in PDT for cancer treatment via type I mechanism (O_2_‐independent).

Superoxide anion radical (O_2_^−•^) produced by charge transfer between the light‐excited PS and adjacent substrates in tumor cells is one of the primary and highly cytotoxic ROS generated from the type I mechanism.^20^, ^21^, ^22^ It reacts with proteins, DNA, or lipids, to achieve irreversible cellular components damage and dysfunction in cell metabolism and even cell apoptosis.^23^ Moreover, under the intracellular superoxide dismutase (SOD)‐mediated disproportionation reactions, O_2_^−•^ molecule further converts to hydrogen peroxide (H_2_O_2_) and O_2_.^24^, ^25^, ^26^ The accumulated H_2_O_2_ further transforms into highly cytotoxic hydroxyl radical (OH•) to significantly aggravate the oxidative damage and enhance PDT anticancer efficiency.^27^, ^28^ Remarkably, O_2_ is partially recyclable during this cascade reaction and valuable to ameliorate the hypoxia microenvironment for enhanced PDT efficacy.^16^, ^29^, ^30^ Thus, O_2_^−•^ generator would be a promising alternative against hypoxic solid tumor treatment.^31^ Inorganic and metallic O_2_^−•^ generators such as TiO_2_ and ZnO generally activated by UV‐light suffer from potential side‐effect and low‐penetration.^32^, ^33^, ^34^, ^35^ Small organic molecular generators undergo water low solubility, and serious aggregation under physiological conditions.^36^, ^37^, ^38^ It still remains a key challenge to develop novel NIR‐trigged O_2_^−•^ generators to circumvent the tumor hypoxia.

Metal–organic framework (MOF), as a new class of hybrid materials, consists of inorganic building units covalently connected by organic building units.^39^, ^40^ Compared to the traditional inorganic/organic nanoparticles, MOF offers adjustable structural and chemical composition at the molecular level together with tunable porosity and chemical stability, which can as transport vehicles for the delivery of imaging agents and biologically active molecules realize accurate diagnosis and treatment of tumors.^41^, ^42^ Porphyrins and porphyrin derivatives as photosensitizers are hydrophobic in nature, which not only cause insufficient selectivity to the site of tumor, but also leads to PS polymerization, reducing the efficacy of PDT and making it very attractive for the assembly structure of organic building units of MOF.^43^ Porphyrin‐based MOF that have been developed so far for potential PDT outcome, however, almost all of them are compounded with other nanoparticles to form new compounds for synergistic treatment (such as PDT‐PTT, PDT‐Radiation therapy),^44^ the complex structures would reduce the clinical medical value.^45^ 2D nanosheets with large surface area, unique physical, and chemical properties have been widely used as theranostic agents for cancer treatment, such as graphene, transitionmetal dichalcogenides, black phosphorus,^46^, ^47^, ^48^ boron nanosheets,^42^ accomplishing multimodal imaging‐guided synergistic treatment. Various 2D nanomaterials were used as nanomedicine for cancer therapy via PDT of type II mechanism in normoxia condition.^49^, ^50^ However, few 2D MOF nanosheets as PDT agents through type I mechanism were developed at present for cancer therapy overcoming the hypoxia.

Herein, we developed a new‐style bacteriochlorin‐based MOFs termed DBBC‐UiO as a NIR laser‐induced O_2_^−•^ generator for photoacoustic imaging (PAI)‐guided PDT through synergistic type I and type II mechanism for hypoxia tumor ablation (**Scheme** 1). The DBBC‐UiO MOF was consisted of 5,15‐di(p‐benzoato)bacteriochlorin (H_2_DBBC) as blocks and heavy Hf_6_(µ_3_‐O)_4_(µ_3_‐OH)_4_ clusters as centers. It is able to act as PS to produce ^1^O_2_ under NIR‐laser irradiation via type II mechanism in adequate O_2_ microenvironment, and also generates considerable O_2_^−•^ via type I mechanism under a severe hypoxic microenvironment with a 750 nm NIR‐laser irradiation. The generated numerous O_2_^−•^ molecules not only act as cytotoxic anion radical to induce tumor cells apoptosis, but also transform to H_2_O_2_ and its downstream highly toxic OH• through SOD‐mediated disproportionation reactions, further promoting the anticancer performance. Meanwhile, the DBBC‐UiO exhibited PAI capability for tumor diagnosis, providing an outstanding strategy for cancer diagnosis with high spatial resolution and deep tissue penetration in clinic. The PAI‐guided striking PDT effect for hypoxic solid tumor ablation under a NIR laser irradiation is promising for clinical hypoxic cancer therapy.

**Scheme 1:**
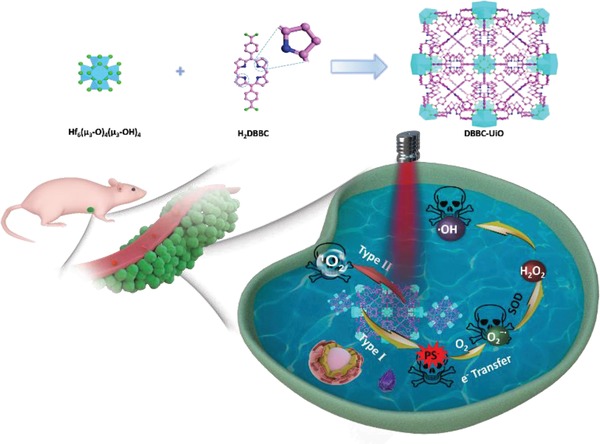
Schematic illustration of the synthetic procedure and photoinduced PDT mechanism of DBBC‐UiO.

The H_2_DBBC block of DBBC‐UiO was prepared by reduction and acidification of 5,15‐di(p‐methyl‐benzoato) porphyrin (Me_2_DBP) as shown in **Figure** 1a, and the nuclear magnetism (^1^HNMR) and mass spectrometry were employed to verify the synthetic procedure and characterize the products in every step (Figures S1–S9, Supporting Information). The transmission electron microscopy (TEM) of DBBC‐UiO MOF revealed a nanosheet morphology with a mean size of about 220 nm and a thickness of 4.6 nm approximately (Figure 1b), and high‐resolution TEM, and fast Fourier transform patterns showed that DBBC‐UiO displayed good crystallinity (inset of Figure 1b). Dynamic light scattering (DLS) results were consistent with the characterization of TEM (Figure 1c). The negligible change of hydrodynamic size for seven consecutive days measurement revealed that DBBC‐UiO MOF presented a good stability in both of PBS (10 × 10^−3^ m, pH 7.4) and biological media (Figure S10, Supporting Information). The power X‐ray diffraction (XRD) of DBBC‐UiO matched with a framework formula of Zr_6_O_4_(OH)_4_(Zn‐DPDBP)_6_, indicating UiO‐type MOF structure that Hf_6_(µ_3_‐O)_4_(µ_3_‐OH)_4_ secondary building units connected with DBBC bridging ligands (Figure 1d; Table S1 and Figures S11 and S12, Supporting Information).^51^ The porosity of DBBC‐UiO measured by nitrogen (N_2_) absorption at 77 K indicated a BET surface area of 198 m^2^ g^−1^ and a pore size of 3.1 nm for DBBC‐UiO (Figure 1e). Encouragingly, the synthetic H_2_DBBC displayed a strong characteristic absorbance peak at the wavelength of 740 nm (Figure S13, Supporting Information). The corresponding peak of the DBBC‐UiO MOF was slightly red‐shifted to 754 nm due to the change of spatial structure and the interaction with Hf^4+^. The strong and broad NIR absorption provided a great potential for PDT with a deeper tumor tissue penetration (Figure 1f). The weaker fluorescence intensity at 822 nm of the DBBC‐UiO compared to H_2_DBBC was resulted from the coordination of the H_2_DBBC ligands to Hf^4+^ ions (Figure 1g), which led to enhanced intersystem crossing (ISC) responsible to ROS generation.^52^ The DBBC‐UiO exhibited a slightly shorter fluorescence lifetime of 8.66 ns compared to 8.80 ns of H_2_DBBC determined by time‐correlated single‐photon counting measurements (Figure 1h).

**Figure 1:**
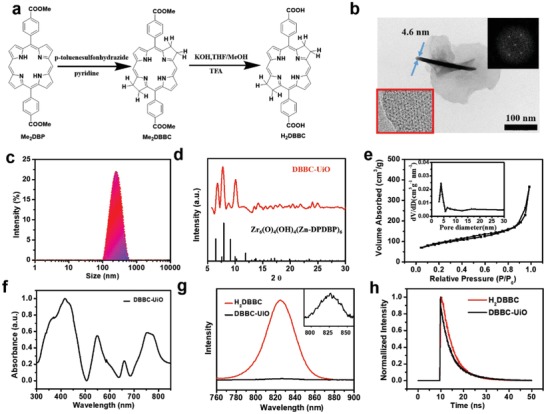
Synthesis and characterizations of H_2_DBBC and DBBC‐UiO. a) Synthetic procedure of H_2_DBBC. b) TEM image, high‐TEM image (insert), and its fast Fourier transform pattern (insert). c) DLS profile of DBBC‐UiO. d) XRD pattern and e) N_2_ adsorption isotherm of DBBC‐UiO, inset in (e): the pore size distribution of DBBC‐UiO. f) UV–vis absorbance of H_2_DBBC and DBBC‐UiO. g) Fluorescence spectra of H_2_DBBC and DBBC‐UiO with excitation at 740 nm, inset in (g): magnified region of DBBC‐UiO from 790 to 860 nm. h) Time‐resolved fluorescence decay traces of H_2_DBBC and DBBC‐UiO.

^1^O_2_ generation efficiency of DBBC‐UiO was first investigated by electron paramagnetic resonance (ESR) using 2,2,6,6‐tetramethylpiperidine (TEMP) as an ^1^O_2_ indicator (**Figure** 2a). The stronger special 1:1:1 triplet characteristic ESR signal assigned to ^1^O_2_ was observed for the DBBC‐UiO after irradiation with a NIR laser, indicating ^1^O_2_ producing ability of the DBBC‐UiO. The ^1^O_2_ luminescence at around 1270 nm that is a characteristic emission peak of ^1^O_2_ further confirmed the ^1^O_2_ generation ability of DBBC‐UiO (Figure 2b). The characteristic absorption of 1,3‐diphenylisobenzofuran (DPBF) decreased continuously with the time going under the NIR irradiation, future indicating that the good production capacity of ^1^O_2_ (Figure 2c; Figure S14, Supporting Information). O_2_^−•^ generation of DBBC‐UiO could also be examined using 5,5‐dimethyl‐1‐pyrroline‐N‐oxide (DMPO) as a probe molecule using ESR spectroscopy. As shown in Figure 2d, an obvious characteristic ESR signal of O_2_^−•^ was observed compared to DBBC‐UiO in dark and only light irradiation, demonstrating the production of O_2_^−•^. However, in the presence of SOD, corresponding ESR signal was sharply decreased, which resulted from SOD‐catalyzed dismutation of O_2_^−•^. Dihydrorhodamine 123 (DHR123) could react with O_2_^−•^ to emit strong green fluorescence with a characteristic peak at 526 nm, which was further applied to monitor the O_2_^−•^ production capability. The DHR123 treated with DBBC‐UiO and irradiated with NIR irradiation exhibited strong green fluorescence related to reaction of O_2_^−•^ and DHR123, while the addition of the SOD and radical scavenger of Vc induced the sharp decrease of the fluorescence (Figure 2e). The fluorescence intensity of DBBC‐UiO under NIR irradiation showed a 3.18‐fold and 3.3‐fold decrease after addition of Vc and SOD, respectively (Figure 2f). These results confirmed the good ^1^O_2_ and O_2_^−•^ generation ability of DBBC‐UiO.

**Figure 2:**
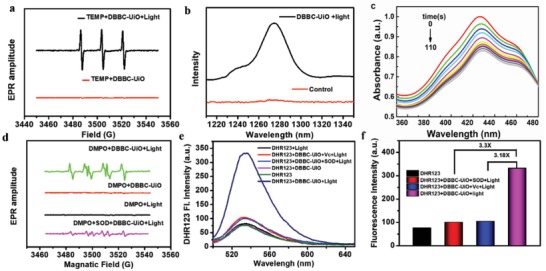
a) ESR spectra of DBBC‐UiO in presence of TEMP with or without NIR‐laser irradiation. b) ^1^O_2_ emission at around 1270 nm induced by DBBC‐UiO with or without NIR‐laser irradiation. c) Time‐course generation of ^1^O_2_ by DBBC‐UiO detecting by the DPBF under NIR laser irradiation. d) ESR signals of DMPO for ^1^O_2_ detection. e) Fluorescence response of DHR 123 in different conditions for O_2_^−•^ detection. f) The corresponding fluorescence intensity in (e).

The cell uptake ability of the resulting DBBC‐UiO MOF nanosheet labeled with FAM was first explored using MCF‐7 as a model. As shown in Figure S15 of the Supporting Information, the DBBC‐UiO was abundant and decentralized in the cells when MCF‐7 cells were exposed to DBBC‐UiO for 4 h, whereas the FAM fluorescence of the lipsome2000 group was comparatively concentrated. This results indicated that the good cell uptake ability of DBBC‐UiO MOF nanosheet, and the high decentralization of the DBBC‐UiO facilitated to the reaction with the substances in living cells, promoting the generation of ROS and accelerating the cell apoptosis. We further examined the ROS generation ability of DBBC‐UiO in living cells under normoxic and hypoxic condition by dihydroethidium (DHE) that react with O_2_^−•^, and the generated product could intercalate with DNA to produce red fluorescence. As shown in **Figure** 3a, the fluorescence intensity enhanced with the increase of the irradiation time, but after Vc treatment, the red fluorescence sharply reduced due to the O_2_^−•^ scavenging effect. Notably, the DHE fluorescence intensity for verifying the O_2_^−•^ generation showed negligible change in MCF‐7 cells with NIR irradiation in the hypoxic condition compared to that in the normoxic condition (Figure 3b; Figure S16, Supporting Information), indicating the PDT effect of DBBC‐UiO MOF under hypoxia. No obvious singlet oxygen sensor green (SOSG) signal associated to ^1^O_2_ was observed in hypoxic condition, whereas numerous ^1^O_2_ was generated in MCF‐7 cells under the normoxic environment (Figure 3b; Figure S16, Supporting Information). 3′‐(4‐hydroxyphenyl) fluorescein (HPF) was used to monitor the OH• generation, and strong fluorescence of HPF was presented in both hypoxic and normoxic conditions after NIR irradiation, which was similar to the O_2_^−•^ (Figure 3b; Figure S16, Supporting Information). Furthermore, SOD inhibitor of 2‐methoxyestradiol was employed to characterize the dismutation. As shown in Figure 3c, under NIR irradiation, the DBBC‐UiO‐treated cells exhibited stronger DHE fluorescence intensity compared to that of the cells only incubated with 2‐methoxyestradiol treatment. Notably, the DBBC‐UiO‐treated cells displayed weaker fluorescence intensity compared to that of DBBC‐UiO‐treated cells received 2‐methoxyestradiol incubation under NIR irradiation. Meanwhile, the addition of 2‐methoxyestradiol induced significant reduction of OH• for cells treated with DBBC‐UiO under NIR irradiation (Figure 3d). These results showed that the inactivation of SOD in cells dramatically inhibited the procession O_2_^−•^ to OH•.

**Figure 3:**
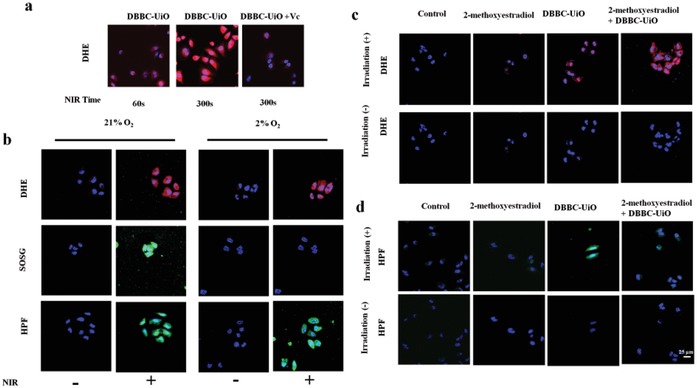
a) Confocal Laser Scanning Microscope (CLSM) images of MCF‐7 cells exposed on NIR laser at different times with or without Vc treatment. b) CLSM images of ROS in DBBC‐UiO‐treated MCF‐7 cells in normoxia and hypoxia environments using DHE, SOSG, and HPF as O_2_^−•^, ^1^O_2_, and •OH detection probe. c) CLSM images of O_2_^−•^ and d) OH• for DBBC‐UiO‐treated MCF‐7 cells treated with or without SOD inhibitor 2‐methoxyestradiol. The scale bar is 25 µm.

The 3‐(4,5‐dimethyl‐2‐thiazolyl)‐2,5‐diphenyl‐2‐H‐tetrazolium bromide (MTT) assay results indicated that the DBBC‐UiO exhibited no appreciable negative effect on the cell viability in absence of NIR‐laser irradiation both in cancer cells (**Figure** 4a) or in normal cells (Figure S17, Supporting Information), suggesting the good biocompatibility of DBBC‐UiO. Neither the PBS (10 × 10^−3^ m, pH 7.4) nor the 750 nm NIR‐laser (1.3 W cm^−2^) irradiation has obvious effect on the cell viability (Figure 4b). On the contrary, the DBBC‐UiO‐treated MCF‐7 cells presented a cell death rate up to 94% with a 750 nm NIR‐laser (1.3 W cm^−2^) irradiation at the conditions of normoxia (21% O_2_) (Figure 4b), which suggested the high anticancer effect. It was worthy to mention that a cell death rate up to 91% was obtained even at the hypoxic condition due to the ROS synergistic effect. As shown in Figure 4c, the Calcien‐AM/PI double staining further suggested the DBBC‐UiO‐transfected cells with NIR‐laser irradiation presented the high cells death rate at the hypoxic (2% O_2_) condition, showing the consistent performance with the MTT. These results suggested the advanced antitumor efficiency of DBBC‐UiO‐mediated PDT.

**Figure 4:**
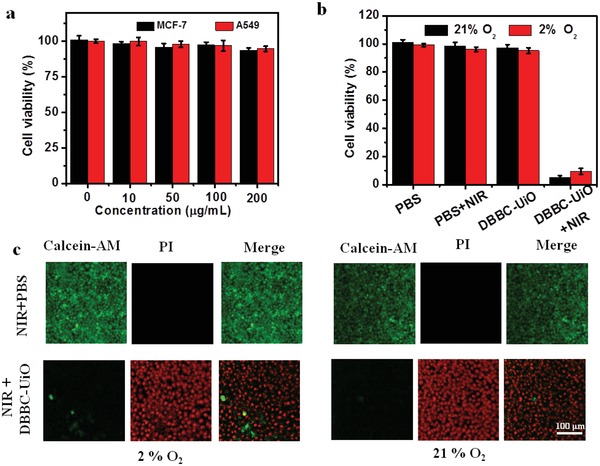
a) Cytotoxicity effect of DBBC‐UiO on MCF‐7 and A549 cells (0–200 µg mL^−1^). b) Relative cell viability of MCF‐7 cells incubated by PBS (10 × 10^−3^ m, pH 7.4) and DBBC‐UiO with/without a 750 NIR‐laser irradiation for 5 min at conditions of 21% O_2_ or 2% O_2_. c) CLSM images of MCF‐7 cells exposed to PBS (10 × 10^−3^ m, pH 7.4) and DBBC‐UiO with a 750 nm NIR‐laser irradiation for 5 min at conditions of 21% O_2_ or 2% O_2_. The scale bar is 100 µm.

The intriguing in vitro antitumor performance encouraged us to further investigate the in vivo performance. As shown in **Figure** 5a, the DBBC‐UiO exhibited good photoacoustic (PA) response, and the concentration‐dependent PA signal intensity increased significantly with the increasing concentration of the DBBC‐UiO (0–1 mg mL^−1^). Because significant tumor accumulation of DBBC‐UiO resulted from the enhanced permeability and retention (EPR) effects, much stronger PA signal could be observed in the tumor tissue after tail vein injection of DBBC‐UiO into mice for 12 h, which confirmed the feasibility of the DBBC‐UiO for in vivo PA imaging (PAI) (Figure 5b). This good PAI capability of the DBBC‐UiO provided a powerful tool for precise tumor tissue diagnosis.

**Figure 5:**
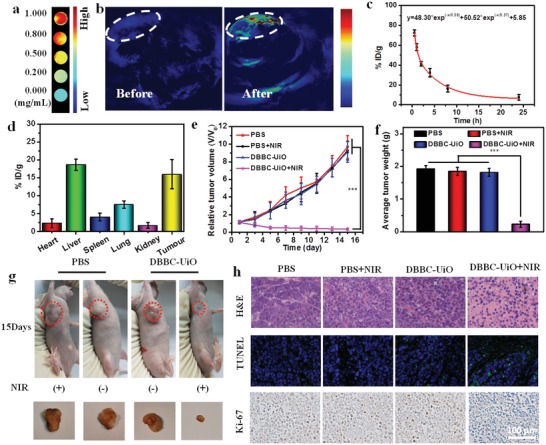
a) The concentration‐dependent PAI of DBBC‐UiO in vitro. b) In vivo PAI with and without injection of DBBC‐UiO. c) Blood circulation curve of DBBC‐UiO in mice through evaluating the concentration of Hf element in the blood at different time points after the injection of DBBC‐UiO. d) Biodistribution of DBBC‐UiO after tail vein injection. The concentration of Hf element was determined by ICP‐AES. Error bars are basis of the standard deviation (SD) among three mice. e) Relative tumor volumes of mice through different treatments. f) Average weights of tumors harvested after 15 d posttreatment. g) Photographs of tumor‐bearing mice received different treatments after 15 d. h) H&E staining, TUNEL staining, and Ki‐67 of tumor sections from different groups. The scale bar is 100 µm.

The metabolism and biodistribution of DBBC‐UiO were further studied. The blood was extracted from the mice post the injection of DBBC‐UiO at desired time points for measuring quantitatively the Hf element concentrations by inductively coupled plasma atomic emission spectrometry (ICP‐AES). It revealed that blood levels of DBBC‐UiO gradually decreased over time according to a two‐compartment model with *t*_1_ = 0.59/*t*_2_ = 5.57 (Figure 5c). The relative long circulation time of DBBC‐UiO in the blood was favorable for effective tumor tissue accumulation (Figure 5c). High tumor accumulation of DBBC‐UiO at ≈13% ID/g was observed after 24 h injection possibly resulted from the tumor EPR effect (Figure 5d). The high levels of accumulation in liver was observed, which suggested DBBC‐UiO in the main organs of mice could decay rapidly over time.

The in vivo PDT antitumor performance of DBBC‐UiO was conducted using nude mice bearing MCF‐7 cells at the right forelimb. It was divided into four groups randomly (*n* = 5) and given tail intravenous injections with PBS (10 × 10^−3^ m, pH 7.4) and DBBC‐UiO (5 mg kg^−1^ for every mouse), respectively, with or without a 750 NIR‐laser (1.3 W cm^−2^) irradiation. The volume of tumor decreased over the time for the mice received treatment of DBBC‐UiO‐injected and the NIR‐laser irradiation, and it almost completely disappeared in 15 d (Figure 5e,g). All the control groups performed a time‐dependent tumor volume increase, showing more than 10‐fold increment compared to the initial volume and no tumor suppression effect (Figure 5e,g). The mice were scarified and the tumors were explanted in 15 d after treatment, and the average tumor weight and size also confirmed the excellent anticancer performance of the DBBC‐UiO‐mediated PDT (Figure 5g,f). The hematoxylin and eosin (H&E) staining revealed the prominent tissue necrosis and numerous cell apoptosis in the tumor tissues for mice treated with the DBBC‐UiO and NIR‐laser irradiation, while no obvious damage was observed for the tumor tissues of other control groups (Figure 5h). Terminal deoxynucleotidyl transferase dUTP nick end‐labeling (TUNEL) assay demonstrated the largest apoptotic cells in the group treated with DBBC‐UiO and NIR among all groups (Figure 5h). The proliferative activity of tumor cells was further analyzed by immunostaining against ki‐67. The DBBC‐UiO‐treated group under NIR‐laser irradiation displayed least ki‐67 positive cells among the all groups (Figure 5h). The H&E staining of the main organs, including heart, spleen, liver, kidney, and lung collected from the mice after different treatments revealed that no obvious damage could be observed in these organs (Figure S18, Supporting Information). These results suggested the good biocompatibility and excellent anticancer efficiency of DBBC‐UiO.

At last, the potential in vivo toxicity of DBBC‐UiO was evaluated by blood routine and biochemical index (Figure 6). As shown in **Figure** 6a, white blood cells (WBC), red blood cells (RBC), hemoglobin (HB), hematocrit (HCT), mean corpuscular hemoglobin (MCH), mean corpuscular hemoglobin concentration (MCHC), platelets (PLT), and mean corpuscular volume (MCV) as the normal hematology parameters were measured at different time point after the mice were treated with tail vein injection of DBBC‐UiO. In general, this monitored markers had no abnormal changes for 14 d compared with control group, indicating that DBBC‐UiO did not cause significant inflammation or infection in the treated mice. The standard blood biochemical indexes were performed and various makers including alanine transaminase (ALT), aspartate transaminase (AST), total protein (TP), globulin (GLOB), albumin/globulin (A/G), blood urea nitrogen (UREA), creatinine (CREA), and albumin (ALB) were examined (Figure 6b). Hence, the DBBC‐UiO treatment had no obviously negative influence compared with control on the blood chemistry. The hepatic or renal related function markers including ALT, AST, UREA in general had no abnormal changes after the mice treated with DBBC‐UiO for a long time, indicating no significant renal and hepatic toxicity in mice. Nevertheless, our results demonstrated that DBBC‐UiO could act as a nanoplatform for clinical cancer therapy with high biocompatibility

**Figure 6:**
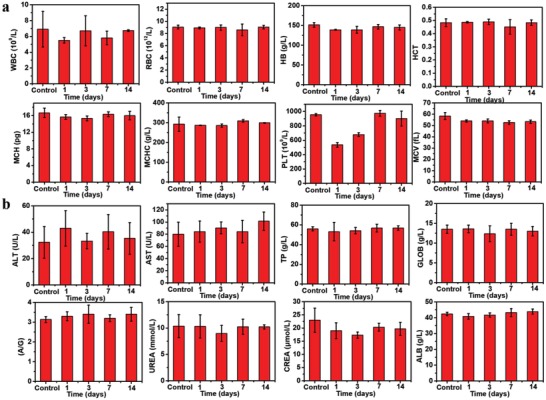
In vivo long‐term toxicity evaluation of DBBC‐UiO. a) Hematological index of the mice including WBC, RBC, HB, HCT, MCH, MCHC, PLT, and MCV. b) Biochemical blood analysis of the mice including ALT, AST, TP, GLOB, A/G, UREA, CREA, and ALB. Untreated healthy mice were used as the control.

In this work, a novel DBBC‐UiO MOF nanosheet was developed as an NIR‐laser triggered O_2_^−•^ generator for PAI‐guided PDT through type I mechanism for selective hypoxia tumor ablation with deep tissue penetration. The DBBC‐UiO enabled to generate abundant O_2_^−•^ within a severe hypoxic microenvironment, and partial O_2_^−•^ converted into high toxic OH• via SOD‐induced catalytic reactions under 750 nm NIR‐laser irradiation, suggesting that the PDT anticancer capacity of DBBC‐UiO was a complete O_2_‐independent procedure for highly efficient hypoxic solid tumor suppression. Tumor‐specific PAI was also verified for cancer accurate diagnosis with deep tissue penetration and high resolution. This work contributed to design a new nanoplatform for specific PDT to overcome the hypoxic cancer microenvironment and light tissue penetration depth for clinical cancer therapy.

## Experimental Section

For detailed experimental conditions and methods of synthesis, and the additional characterizations, see Figures S1–S14 (Supporting information). All animal experiments were performed according to protocols approved by the Department of Laboratory Animal Science at Peking University Health Science Center.

## Conflict of Interest

The authors declare no conflict of interest.

## Supporting information

Supplementary

## References

[1] J. M. Brown , W. R. Wilson , Nat. Rev. Cancer 2004, 4, 437. 15170446 10.1038/nrc1367

[2] J. E. Moulder , S. Rockwell , Cancer Metastasis Rev. 1987, 5, 313. 3552280 10.1007/BF00055376

[3] W. Piao , K. Hanaoka , T. Fujisawa , S. Takeuchi , T. Komatsu , T. Ueno , T. Terai , T. Tahara , T. Nagano , Y. Urano , J. Am. Chem. Soc. 2017, 139, 13713. 28872304 10.1021/jacs.7b05019

[4] T. J. Dougherty , C. J. Gomer , B. W. Henderson , G. Jori , D. Kessel , M. Korbelik , J. Moan , Q. Peng , JNCI J. Natl. Cancer Inst. 1998, 90, 889. 9637138 10.1093/jnci/90.12.889 PMC4592754

[5] X. Li , B.‐D. Zheng , X.‐H. Peng , S.‐Z. Li , J.‐W. Ying , Y. Zhao , J.‐D. Huang , J. Yoon , Coord. Chem. Rev. 2019, 379, 147.

[6] J. P. Celli , B. Q. Spring , I. Rizvi , C. L. Evans , K. S. Samkoe , S. Verma , B. W. Pogue , T. Hasan , Chem. Rev. 2010, 110, 2795. 20353192 10.1021/cr900300p PMC2896821

[7] A. P. Castano , P. Mroz , M. R. Hamblin , Nat. Rev. Cancer 2006, 6, 535. 16794636 10.1038/nrc1894 PMC2933780

[8] X. Li , S. Yu , D. Lee , G. Kim , B. Lee , Y. Cho , B.‐Y. Zheng , M.‐R. Ke , J.‐D. Huang , K. T. Nam , X. Chen , J. Yoon , ACS Nano 2018, 12, 681. 29232105 10.1021/acsnano.7b07809

[9] W. Fan , P. Huang , X. Chen , Chem. Soc. Rev. 2016, 45, 6488. 27722560 10.1039/c6cs00616g

[10] S. Wang , F. Yuan , K. Chen , G. Chen , K. Tu , H. Wang , L. Q. Wang , Biomacromolecules 2015, 16, 2693. 26207413 10.1021/acs.biomac.5b00571

[11] C. Liu , H. Dong , N. Wu , Y. Cao , X. Zhang , ACS Appl. Mater. Interfaces 2018, 10, 6991. 29405051 10.1021/acsami.8b00112

[12] R. Song , D. Hu , H. Y. Chung , Z. Sheng , S. Yao , ACS Appl. Mater. Interfaces 2018, 10, 36805. 30300545 10.1021/acsami.8b15293

[13] X. Song , L. Feng , C. Liang , K. Yang , Z. Liu , Nano Lett. 2016, 16, 6145. 27622835 10.1021/acs.nanolett.6b02365

[14] Z. Zhou , B. Zhang , S. Wang , W. Zai , A. Yuan , Y. Hu , J. Wu , Small 2018, 14, 1801694. 10.1002/smll.201801694 30307696

[15] S.‐Y. Li , H. Cheng , B.‐R. Xie , W.‐X. Qiu , J.‐Y. Zeng , C.‐X. Li , S.‐S. Wan , L. Zhang , W.‐L. Liu , X.‐Z. Zhang , ACS Nano 2017, 11, 7006. 28665106 10.1021/acsnano.7b02533

[16] Y. Zhang , F. Wang , C. Liu , Z. Wang , L. Kang , Y. Huang , K. Dong , J. Ren , X. Qu , ACS Nano 2018, 12, 651. 29290107 10.1021/acsnano.7b07746

[17] H. Chen , J. Tian , W. He , Z. Guo , J. Am. Chem. Soc. 2015, 137, 1539. 25574812 10.1021/ja511420n

[18] H. Fan , G. Yan , Z. Zhao , X. Hu , W. Zhang , H. Liu , X. Fu , T. Fu , X.‐B. Zhang , W. Tan , Angew. Chem., Int. Ed. 2016, 55, 5477. 10.1002/anie.201510748 PMC4971833 27010667

[19] W. Zhang , S. Li , X. Liu , C. Yang , N. Hu , L. Dou , B. Zhao , Q. Zhang , Y. Suo , J. Wang , Adv. Funct. Mater. 2018, 28, 1706375.

[20] M. Ushio‐Fukai , Y. Nakamura , Cancer Lett. 2008, 266, 37. 18406051 10.1016/j.canlet.2008.02.044 PMC2673114

[21] E. G. Ju , K. Dong , Z. W. Chen , Z. Liu , C. Q. Liu , Y. Y. Huang , Z. Z. Wang , F. Pu , J. S. Ren , X. G. Qu , Angew. Chem., Int. Ed. 2016, 55, 11467. 10.1002/anie.201605509 27504861

[22] Y. Cheng , Y. Chang , Y. L. Feng , N. Liu , X. J. Sun , Y. Q. Feng , X. Li , H. Y. Zhang , Small 2017, 13, 1603935.

[23] P. a. Ma , H. Xiao , C. Yu , J. Liu , Z. Cheng , H. Song , X. Zhang , C. Li , J. Wang , Z. Gu , J. Lin , Nano Lett. 2017, 17, 928. 28139118 10.1021/acs.nanolett.6b04269

[24] J. Wu , X. Wang , Q. Wang , Z. Lou , S. Li , Y. Zhu , L. Qin , H. Wei , Chem. Soc. Rev. 2019, 48, 1004. 30534770 10.1039/c8cs00457a

[25] Y. Liu , K. Ai , X. Ji , D. Askhatova , R. Du , L. Lu , J. Shi , J. Am. Chem. Soc. 2017, 139, 856. 27997170 10.1021/jacs.6b11013 PMC5752099

[26] Z. Yang , Y. Dai , C. Yin , Q. Fan , W. Zhang , J. Song , G. Yu , W. Tang , W. Fan , B. C. Yung , J. Li , X. Li , X. Li , Y. Tang , W. Huang , J. Song , X. Chen , Adv. Mater. 2018, 30, e1707509. 29707841 10.1002/adma.201707509

[27] M. Huo , L. Wang , Y. Chen , J. Shi , Nat. Commun. 2017, 8, 357. 28842577 10.1038/s41467-017-00424-8 PMC5572465

[28] W. Zhen , Y. Liu , L. Lin , J. Bai , X. Jia , H. Tian , X. Jiang , Angew. Chem., Int. Ed. 2018, 57, 10309. 10.1002/anie.201804466 29888846

[29] J. Kim , H. R. Cho , H. Jeon , D. Kim , C. Song , N. Lee , S. H. Choi , T. Hyeon , J. Am. Chem. Soc. 2017, 139, 10992. 28737393 10.1021/jacs.7b05559

[30] M. S. Wason , J. Colon , S. Das , S. Seal , J. Turkson , J. Zhao , C. H. Baker , Nanomedicine 2013, 9, 558. 23178284 10.1016/j.nano.2012.10.010 PMC3606274

[31] M. Li , J. Xia , R. Tian , J. Wang , J. Fan , J. Du , S. Long , X. Song , J. W. Foley , X. Peng , J. Am. Chem. Soc. 2018, 140, 14851. 30362735 10.1021/jacs.8b08658

[32] Y. Liu , X. Meng , H. Wang , Z. Tang , C. Zuo , M. He , W. Bu , ACS Appl. Mater. Interfaces 2018, 10, 1492. 29271197 10.1021/acsami.7b14451

[33] G. Liu , W. Li , R. Bi , C. Atangana Etogo , X.‐Y. Yu , L. Zhang , ACS Catal. 2018, 8, 1720.

[34] P. A. Pepin , J. D. Lee , C. B. Murray , J. M. Vohs , ACS Catal. 2018, 8, 11834.

[35] H. Zhang , W. Wang , H. Zhao , L. Zhao , L.‐Y. Gan , L.‐H. Guo , ACS Catal. 2018, 8, 9399.

[36] H. Chen , Z. Gu , H. An , C. Chen , J. Chen , R. Cui , S. Chen , W. Chen , X. Chen , X. Chen , Z. Chen , B. Ding , Q. Dong , Q. Fan , T. Fu , D. Hou , Q. Jiang , H. Ke , X. Jiang , G. Liu , S. Li , T. Li , Z. Liu , G. Nie , M. Ovais , D. Pang , N. Qiu , Y. Shen , H. Tian , C. Wang , H. Wang , Z. Wang , H. Xu , J.‐F. Xu , X. Yang , S. Zhu , X. Zheng , X. Zhang , Y. Zhao , W. Tan , X. Zhang , Y. Zhao , Sci. China: Chem. 2018, 61, 1503.

[37] Z. Yang , Y. Dai , L. Shan , Z. Shen , Z. Wang , B. C. Yung , O. Jacobson , Y. Liu , W. Tang , S. Wang , L. Lin , G. Niu , P. Huang , X. Chen , Nanoscale Horiz. 2019, 4, 426. 10.1039/C8NH00307F PMC6764780 31565239

[38] Y. Zhen , F. Wenpei , T. Wei , S. Zheyu , D. Yunlu , S. Jibin , W. Zhantong , L. Yuan , L. Lisen , S. Lingling , Angew. Chem., Int. Ed. 2018, 57, 14101.

[39] S. Wuttke , M. Lismont , A. Escudero , B. Rungtaweevoranit , W. J. Parak , Biomaterials 2017, 123, 172. 28182958 10.1016/j.biomaterials.2017.01.025

[40] P. Hirschle , T. Preiß , F. Auras , A. Pick , J. Völkner , D. Valdepérez , G. Witte , W. J. Parak , J. O. Rädler , S. Wuttke , CrystEngComm 2016, 18, 4359.

[41] T. Simon‐Yarza , A. Mielcarek , P. Couvreur , C. Serre , Adv. Mater. 2018, 30, 1707365. 10.1002/adma.201707365 29876985

[42] X. Ji , N. Kong , J. Wang , W. Li , Y. Xiao , S. T. Gan , Y. Zhang , Y. Li , X. Song , Q. Xiong , S. Shi , Z. Li , W. Tao , H. Zhang , L. Mei , J. Shi , Adv. Mater. 2018, 30, 1803031. 10.1002/adma.201803031 PMC6338531 30019786

[43] M. Yu , L. Xiangyuan , L. Aijie , Y. Peng , Z. Caiyun , T. Bo , Angew. Chem., Int. Ed. 2017, 56, 13752.

[44] W. Cheng , J. Nie , N. Gao , G. Liu , W. Tao , X. Xiao , L. Jiang , Z. Liu , X. Zeng , L. Mei , Adv. Funct. Mater. 2017, 27, 1704135.

[45] R. Freund , U. Lachelt , T. Gruber , B. Ruhle , S. Wuttke , ACS Nano 2018, 12, 2094. 29533060 10.1021/acsnano.8b00932

[46] X. Liang , X. Ye , C. Wang , C. Xing , Q. Miao , Z. Xie , X. Chen , X. Zhang , H. Zhang , L. Mei , J. Controlled Release 2019, 296, 150. 10.1016/j.jconrel.2019.01.027 30682441

[47] W. Tao , X. Zhu , X. Yu , X. Zeng , Q. Xiao , X. Zhang , X. Ji , X. Wang , J. Shi , H. Zhang , L. Mei , Adv. Mater. 2017, 29, 1603276. 10.1002/adma.201603276 PMC5205548 27797119

[48] X. Zeng , M. Luo , G. Liu , X. Wang , W. Tao , Y. Lin , X. Ji , L. Nie , L. Mei , Adv. Sci. 2018, 5, 1800510. 10.1002/advs.201800510 PMC6193171 30356942

[49] J. Wen , K. Yang , F. Liu , H. Li , Y. Xu , S. Sun , Chem. Soc. Rev. 2017, 46, 6024. 28848978 10.1039/c7cs00219j

[50] Q. Chen , J. Wen , H. Li , Y. Xu , F. Liu , S. Sun , Biomaterials 2016, 106, 144. 27561885 10.1016/j.biomaterials.2016.08.022

[51] K. Lu , C. He , W. Lin , J. Am. Chem. Soc. 2015, 137, 7600. 26068094 10.1021/jacs.5b04069 PMC4691355

[52] K. Lu , C. He , W. Lin , J. Am. Chem. Soc. 2014, 136, 16712. 25407895 10.1021/ja508679h PMC4277757

