# Supplementary material for: A Bacteriochlorin‐Based Metal–Organic Framework Nanosheet Superoxide Radical Generator for Photoacoustic Imaging‐Guided Highly Efficient Photodynamic Therapy
Source: Adv Sci (Weinh). 2019 May 16;6(14):1900530. doi: 10.1002/advs.201900530 (PMC6661935; doi:10.1002/advs.201900530)
Supplement: Supplementary file 1 — Supplementary [file ADVS-6-1900530-s001.pdf]

Copyright WILEY-VCH Verlag GmbH & Co. KGaA, 69469 Weinheim, Germany, 2019.

ADVANCED SCIENCE
Open Access

Supporting Information

for *Adv. Sci.,* DOI: 10.1002/advs.201900530

A Bacteriochlorin-Based Metal–Organic Framework Nanosheet Superoxide Radical Generator for Photoacoustic Imaging-Guided Highly Efficient Photodynamic Therapy

Kai Zhang, Zhaofeng Yu, Xiangdan Meng, Weidong Zhao, Zhuojie Shi, Zhou Yang,* Haifeng Dong,* and Xueji Zhang*

A Bacteriochlorin-Based Metal-Organic Framework Nanosheet Superoxide Radical Generator for Photoacoustic Imaging-Guided Highly Efficient Photodynamic Therapy

Kai Zhang,[a,c] Zhaofeng Yu,[a] Xiangdan Meng,[a,b,]* Weidong Zhao,[a] Zhuojie Shi,[a] Zhou Yang,[a,]* Haifeng Dong,[b,]* Xueji Zhang[b]

[a] School of Materials Science and Engineering, University of Science & Technology Beijing, 30 Xueyuan Road, Beijing 100083 (P.R. China)

E-mail: mengxiangdan1990@163.com

E-mail: yangz@ustb.edu.cn

[b] Beijing Key Laboratory for Bioengineering and Sensing Technology, Research Center for Bioengineering and Sensing Technology, School of Chemistry & Biological Engineering, University of Science & Technology Beijing, 30 Xueyuan Road, Beijing 100083 (P.R. China)

E-mail: hfdong@ustb.edu.cn

[c] Tianjin Key Laboratory of Radiation Medicine and Molecular Nuclear Medicine, Institute of Radiation Medicine, Chinese Academy of Medical Science and Peking Union Medical College, Tianjin 300192 (P.R. China)

Materials: All the chemical reagents and solvents in our research were of analytical grade and used upon receipt without further purification. Paraformaldehyde, pyrrole, trifluoroacetic acid, hafnium (IV) chloride and p-Toluenesulfonyl hydrazide was obtained from Energy Chemical Co., Ltd. (Beijing, China). 5,5-Dimethyl-1-pyrroline n-oxide (DMPO), tetramethylpiperidine (TEMP), tetramethylpiperidinooxy (TEMPO), 3'-(4-hydroxyphenyl) fluorescein (HPF) and dihydrorhodamine 123 (DHR123) were purchased form Sigma-Aldrich Co., Ltd. (St. Louis, USA). Hoechst 33342, dihydroethidium (DHE) and 3-(4,5-dimethylthiozol-2-yl)-2,5-diphenyltetrazolium bromide (MTT) were provided by Beyotime Biotechnology Co., Ltd. (Shanghai, China). Fetal bovine serum (FBS), Dulbecco's modified Eagle's medium (DMEM), penicillin-streptomycin (PS) and singlet oxygen sensor green (SOSG) were provided by Life Technologies Corporation (Los Angeles, USA)

Instruments: The ^{1}H NMR spectra was characterized by a Bruker Dmx500 MHz spectrometer (Switzerland) at room temperature. The mass spectra (MS) was determined by a Bruker Autoflex-3 smart beam mass spectrometer with MALDI-TOF. The Micro-morphologies of DBBC-UiO were obtained by a transmission electron microscope (TEM, JEM-2000FX). The X-ray diffraction (XRD) analysis was measured on with a Cu-Kα radiation (λ=0.15418 nm). The UV-vis absorption spectra was acquired by using a JASCO V-570 spectrophotometer (JASCO, Japan), and the Fourier Transform Infrared (FT-IR) spectroscopy was examined in a PerkinElmer spectrum by preparing sample pellets with KBr. The zeta potential and dynamic light scattering (DLS) analysis were performed through a Zetasizer Nano-ZS ZEN3600 instrument (Malvern Instruments, UK). The transient absorption spectra and fluorescence spectra were carried on a Edinburgh Analytical Instruments FLS980 lifetime and steady-state spectrometer. Confocal laser scanning microscope (CLSM) images were acquired by Olympus FV1200 confocal laser scanning microscope. The photoacoustic imaging (PAI) in vitro and in vivo were acquired from a MOST inVision 128 imaging system (iThera Medical, Germany).

Synthesis of 2-pyrrolemethane: Paraformaldehyde (1.50 g) was dissolved in excess distilled pyrrole (150 mL), where pyrrole was not only a reactant but also a reaction solvent. The solution was placed in a 500 mL two-necked round bottom flask and protected from light, heated and stirred at 55 °C to dissolve most of the solid under nitrogen protection. Then anhydrous indium trichloride was added (2.93 g, 10.0 mmol) at 55 °C and stirring for 2.5 hours, then sodium hydroxide (0.50 g, 40.0 mmol) was added and stirring was continued for half an hour. The excess pyrrole was distilled off under reduced pressure. The product was purified by column chromatography using a mixed solvent of ethyl acetate: petroleum ether = 1:8 (v / v) as the eluent to afford white the flocculent solid compound.

Synthesis of 5,15-di(p-methyl-benzoato) porphyrin (Me_2DBP): 4-(Methoxycarbonyl) benzaldehyde (1.20 g, 7.3 mmol) and dipyrrylmethane (1.07 g, 7.3 mmol) were dissolved in anhydrous dichloromethane (DCM) under N_2 and protected from light. To the reaction mixture was added Trifluoroacetic acid (0.34 mL, 4.4 mmol) dropwise and stirred at room temperature for 4 hours. To the reaction mixture was added 2.49 g of 2,3-dichloro-5,6-dicyano-1,4-benzoquinone (DDQ, 11.0 mmol), followed by further stirring for 1 hour. Triethylamine was added to neutralize the reaction mixture. The solvent was removed on a rotary evaporator. The 5,15-bis (p-toluic acid) porphyrin (Me_2DBP) product was purified by column chromatography using DCM as eluent. Yield: 810 mg, 1.40 mmol (38%). ^{1}H-NMR (500 MHz, chloroform-D, ppm): δ=10.39 (s, 2H), 9.45 (d, 4H), 9.08 (d, 4H), 8.54 (d, 4H),8.39 (d, 4H), 4.17 (s, 6H), -3.10 (brs, 2H). MALDI-TOF MS: m/z calcd for $C_{36}H_{26}N_4O_4$ 578.2, found 578.3.

Synthesis of Me_2DBBC: Under N_2 atmosphere, 30 ml anhydrous pyridine was added to a 2-neck round bottom flask containing Me_2DBP (128mg, 0.22mmol), p-Toluenesulfonyl hydrazide (0.2g, 1.1 mmol) and anhydrous K_2CO_3 (1.5g). The reaction was stirring at 110 ℃ in dark. Meanwhile, 12.8 ml p-toluenesulfonylhydrazine (0.8M in anhydrous pyridine) was added into the above solution at the speed of 0.8 mL/h, then stirring for further 4 h. UV detection was used determine the generation of

bacteriochlorin (band at 736 nm) throughout the entire reaction. After reaction, the mixture was added to EtOAc/H_2O (2:1) and heated to reflux for 2 h. When cooling to room temperature, the organic phase washed by 2 M HCl, 8 M phosphoric, saturated $NaHCO_3$ solution and water. Finally, the dark green solid product Me_2DBBC was collected by vacuum filtration and freeze-drying with a yield of 65%. 1H-NMR (500 MHz, chloroform-D, ppm): δ=8.78 (s, 2H), 8.56 (d, 2H), 8.37 (d, 4H), 8.09 (d, 2H), 7.97 (d, 4H), 4.50 (t, 4H), 4.16 (t, 4H), 4.08 (s, 6H), -1.57 (s, 2H). MALDI-TOF MS: m/z calcd for $C_{36}H_{30}N_4O_4$ 582.2, found 582.4.

Synthesis of H_2DBBC: Me_2DBBC (40 mg, 0.1 mmol) dissolved in a two-necked flask containing 20 mL of solution (V_{THF}/$V_{methanol}$ =1:1) deoxidize for 30 minutes under N_2 atmosphere and in dark. An aqueous KOH solution (2 mol/L, 40 equiv.) was added gradually into the above solution and heated to reflux overnight. The solvent was removed by a rotary evaporator and then 10 ml of deionized water (DI) was added, trifluoroacetic acid was then added dropwise until it was adjusted to pH=3. The dark red solid was collected by vacuum filtration, washed several times with DI and diethyl ether until the filtrate was colorless, and the product of H_2DBBC with a yield of 70% was obtained by freeze-drying. 1H-NMR (500 MHz, Trifluoroacetic acid-D, ppm): δ=8.89 (s, 2H), 8.59 (d, 4H), 8.49 – 8.44 (m, 2H), 8.21 (d, 2H), 8.12 (d, 2H), 7.96 – 7.89 (m, 2H), 4.72 – 4.60 (m, 4H), 4.58 – 4.46 (m, 4H). MALDI-TOF MS: m/z calcd for $C_{34}H_{26}N_4O_4$ 554.2, found 554.5.

Preparation of DBBC-UiO: DBBC-UiO was prepared according to the previous literature. H_2DBBC (3.5 mg, 0.006 mmol) was dissolved in 1 mL of N,N-dimethylformamide (DMF), and $HfCl_4$ (2 mg, 0.006 mmol) was dissolved in 1 mL of DMF and 0.15 mL of acetic acid (2.6 mmol). The two above solutions were mixed in a glass vial and kept at 90°C for three days. After cooling down to room temperature, the product was obtained by centrifugation and washed with DMF, triethylamine/ethanol (the volume ratio of 1:20) solution and ethanol three times, respectively.

Superoxide anion radical ($O_2^{-\bullet}$) detection: DHR123 was used as an $O_2^{-\bullet}$ indicator, which could be converted to Rhodamine 123 in the presence of $O_2^{-\bullet}$. Both the DBBC-UiO and DHR123 were prepared as a 5 μM aqueous solution. Fluorescence spectra was measured immediately after the NIR laser irradiation. For $O_2^{-\bullet}$ quenching experiment, 10 μM of Vc or SOD were added into the above mixture solution before NIR laser irradiation.

Hydroxyl radical (OH•) detection: For OH• evaluation, the experiment procedure was same as $O_2^{-\bullet}$ measurement except using the HPF as the OH• indicator.

Singlet oxygen ($^1O_2$) generation: $^1O_2$ generation was determined by using a $^1O_2$ capture agent SOSG which could capture and react with $^1O_2$ molecule rapidly.

Intracellular ROS detection: Before MCF-7 cells were incubated with 10 μM fluorescence indicator for further 30 min, MCF-7 cells were exposed to DBBC-UiO for 4 h. Then, the cells were irradiated with NIR light for different time. The fluorescence imaging was observed immediately through CLSM.

Cell culturing: MCF-7 cells were seeded in cell culture plates (10 mm) at 37 ^oC under 5% CO_2 atmosphere in DMEM supplemented with 10% FBS and 2% PS. Additionally, in order to mimic the hypoxic tumor microenvironment, MCF-7 cells were cultured in an incubator chamber (MIC-101, Billups-rothenberg) at 37^oC under a humidified atmosphere containing 2% O_2 and 5% CO_2 and used an oxygen detector (Nuvair, O_2 Qucikstick) to monitor the oxygen content in the chamber. Other operations were same to that in normoxic environment.

Cell cytotoxicity assay: To evaluate the therapy efficiency of the resultant photosensitizer (DBBC-UiO), the cells toxicity in vitro was first performed. MCF-7 cells were co-cultured with Opti-MEM containing different concentrations of DBBC-UiO for 4 h. Then, the solution was removed and washed the cells with PBS three times, and the cells were incubated for another 24 h before tested by the methyl thiazolyl terazolium (MTT).

In vitro therapy performance: MCF-7 cells were co-incubated in a Opti-MEM media with a concentration of DBBC-UiO at 50 μg/mL or PBS (10 mM, pH 7.4) for 4 h, respectively. Then the cells were washed by PBS (10 mM, pH 7.4), and re-suspended in a 1.5 mL tube with different treatments. The treated MCF-7 cells were immediately transferred into a 96-well plate and incubated for another 24 h before standard MTT experiments operation.

Animal experiments: According to protocols approved by the Department of Laboratory Animal Science at Peking University Health Science Center, female nude mice with body weights about 20 g were purchased from Beijing Weitong Lihua Experimental Animal Technology Co. Ltd, chosen as the allograft tumor model. Tumors of mice were prepared by injected 100 μL of PBS (10 mM, pH 7.4) containing 10^6 MCF-7 cells into the mice, PAI and photodynamic therapy (PDT) effect were performed when tumor size reached about 50-60 mm^3 large.

For in Vivo PA imaging: 200 μL of DBBC-UiO (1 mg/mL) or PBS (10 mM, pH 7.4) were injected into the tumor-bearing mice through the tail vein. PA imaging was performed through a MOST inVision 128 imaging system after the injection of 12 h.

In Vivo PDT Effect: The tumor-bearing nude mice were divided randomly to four groups (n = 5 per group) and then different treatments were performed. (1) Control group, animals only received PBS (100 μL, 10 mM, pH 7.4) injection through the tail vein; (2) PBS (100 μL, 10 mM, pH 7.4)+NIR; (3) DBBC-UiO (5.0 mg/kg) only; (4) DBBC-UiO (5.0 mg/kg) + NIR. 4 h later, the tumors were received a 740 nm NIR laser (1.3 W cm^{-2}) irradiation for 5 min and the laser irradiation was repeated two times within a 10 min interval. The volumes of tumor were monitored daily by a digital caliper for 15 days and normalized against their initial sizes (0 days). Two days after treatment, the mice from each groups were sacrificed, and the tumors and other major organs (including heart, liver, kidney, spleen and lung) were explanted and collected for anatomy and histo-pathological analysis.

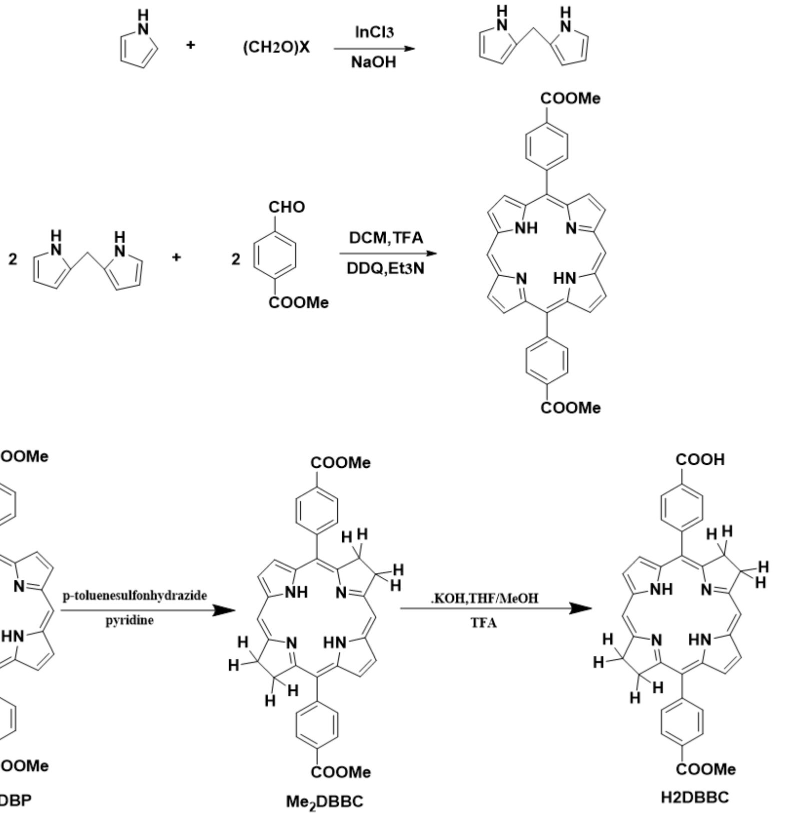

Scheme S1. The main synthesis routes.

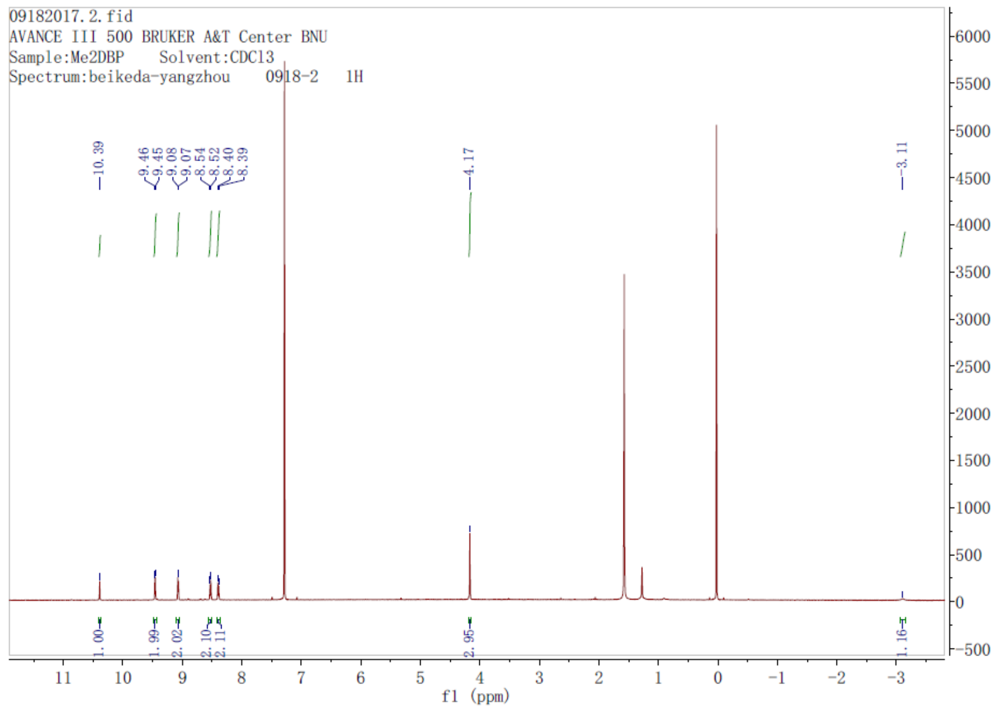

Figure S1. ^1H NMR spectrum of Me_2DBP in chloroform-D.

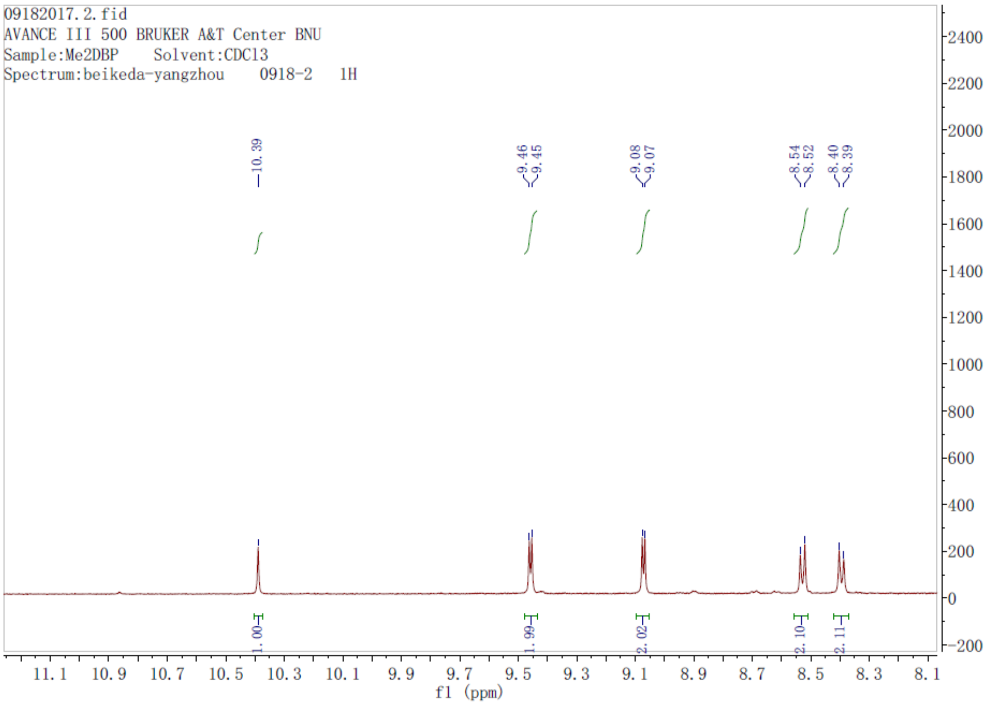

Figure S2. The amplified view of a portion of Figure S1.

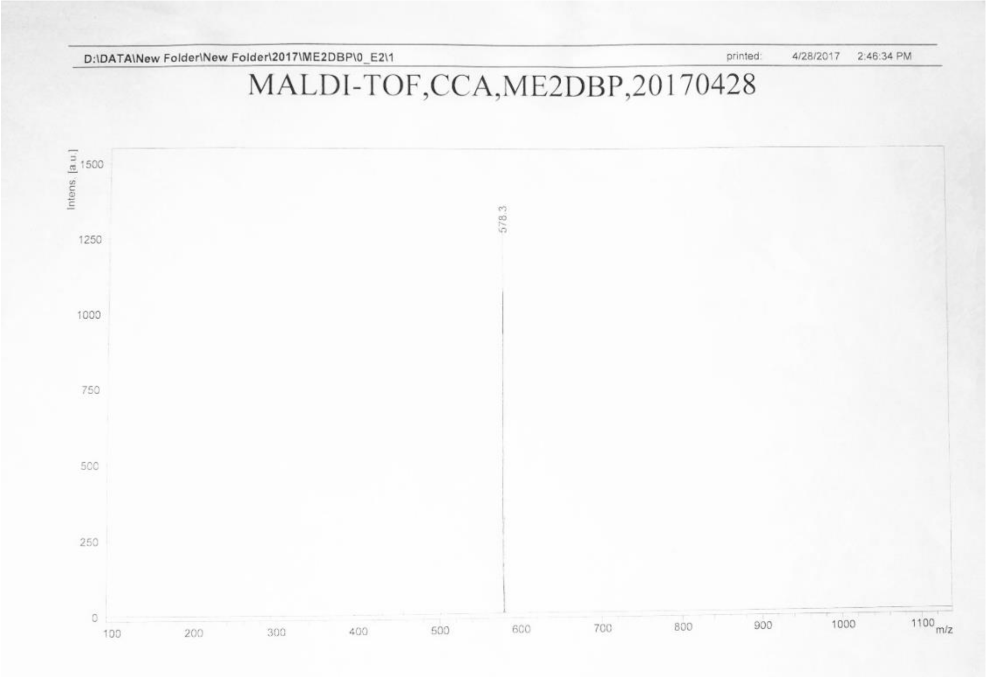

Figure S3. MALDI-TOF MS of Me_2DBP.

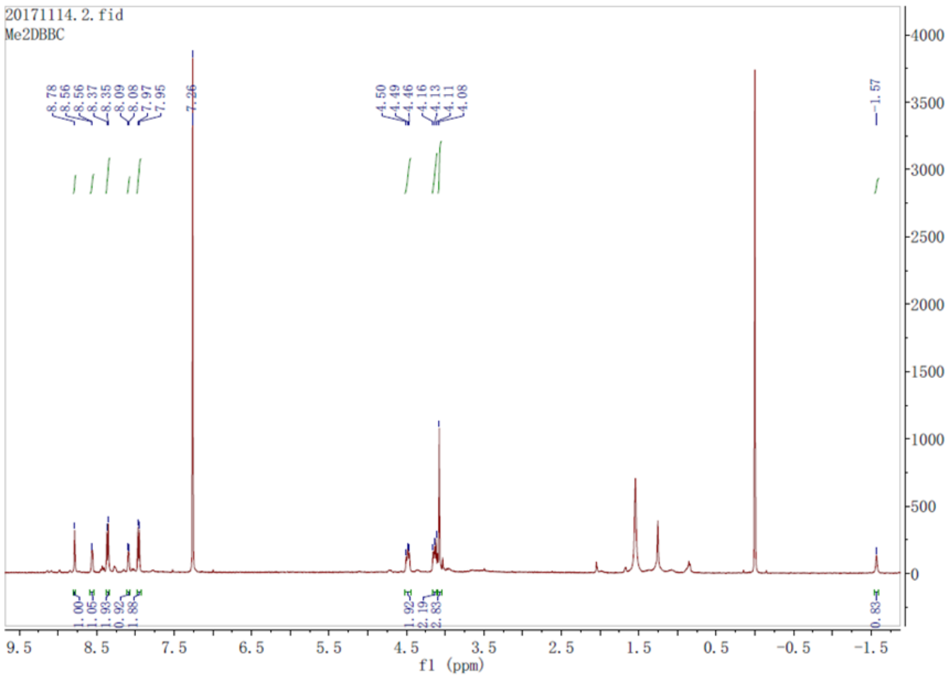

Figure S4. ^{1}H NMR spectrum of Me_2DBBC in chloroform-D.

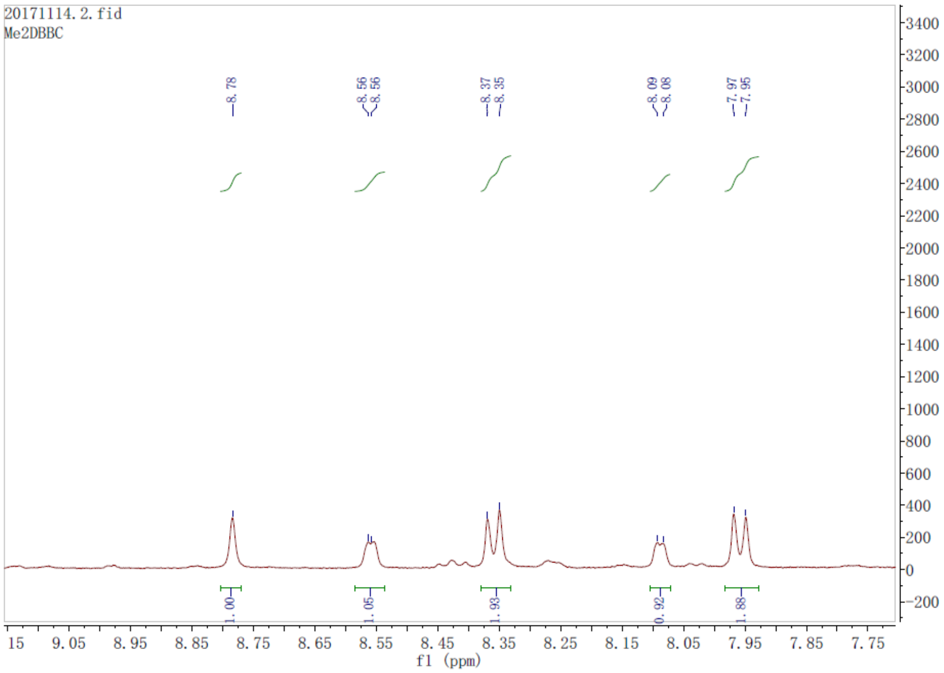

Figure S5. The amplified view of a portion of Figure S4.

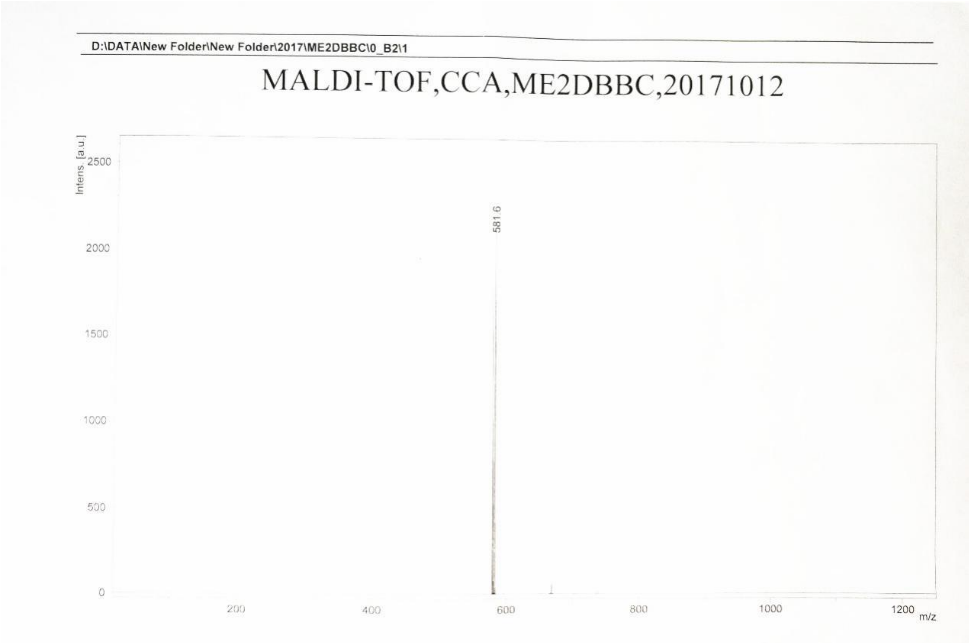

Figure S6. MALDI-TOF MS of Me_2DBBC.

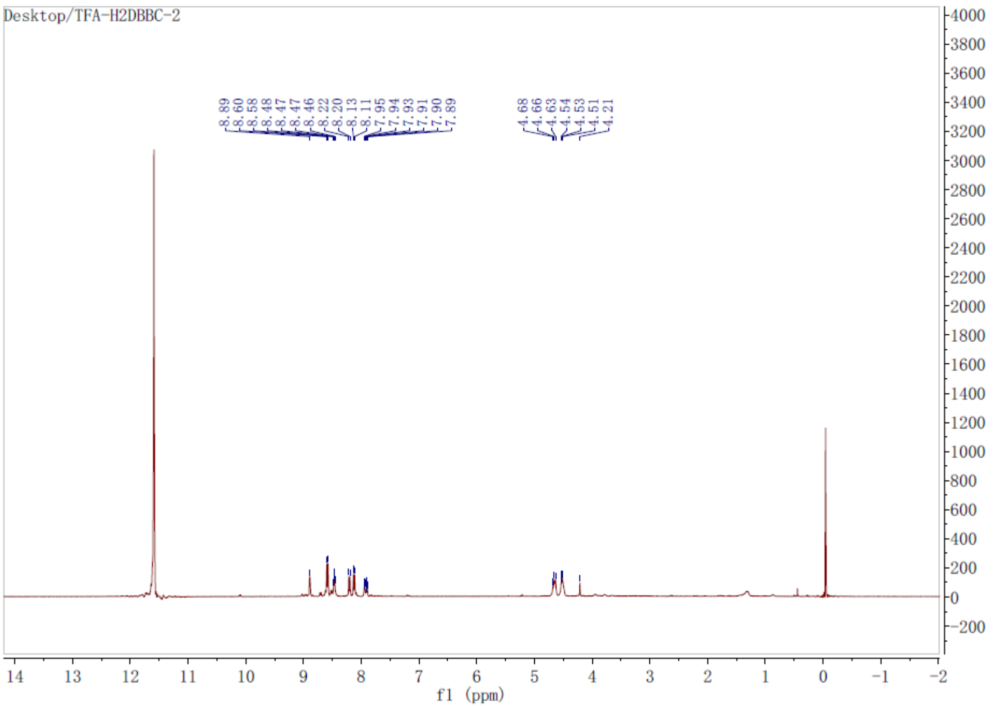

Figure S7. ^{1}H NMR spectrum of H_2DBBC in Trifluoroacetic acid-D.

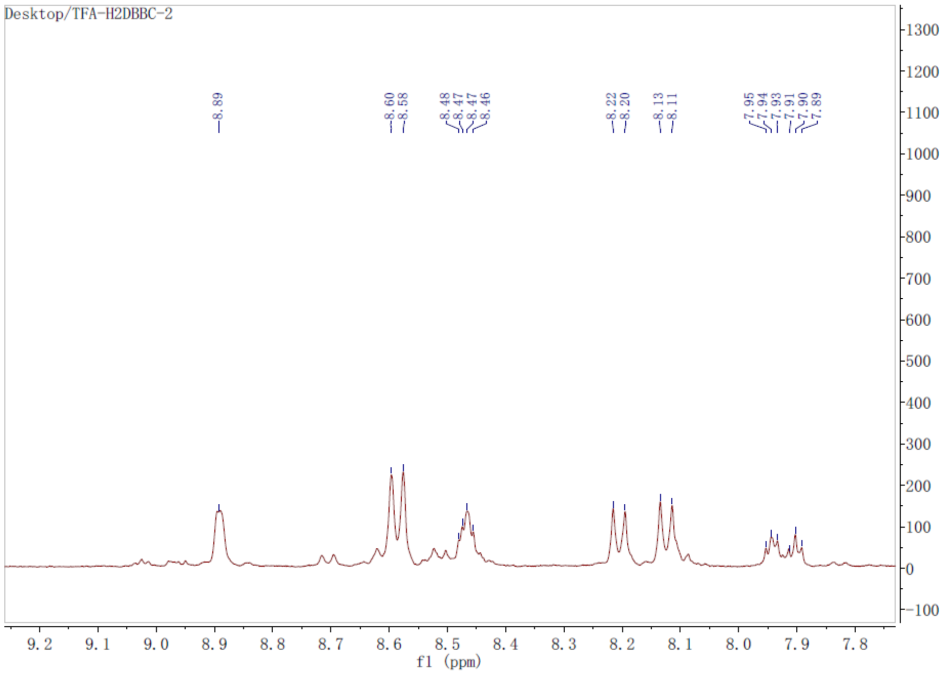

Figure S8. The amplified view of a portion of Figure S7.

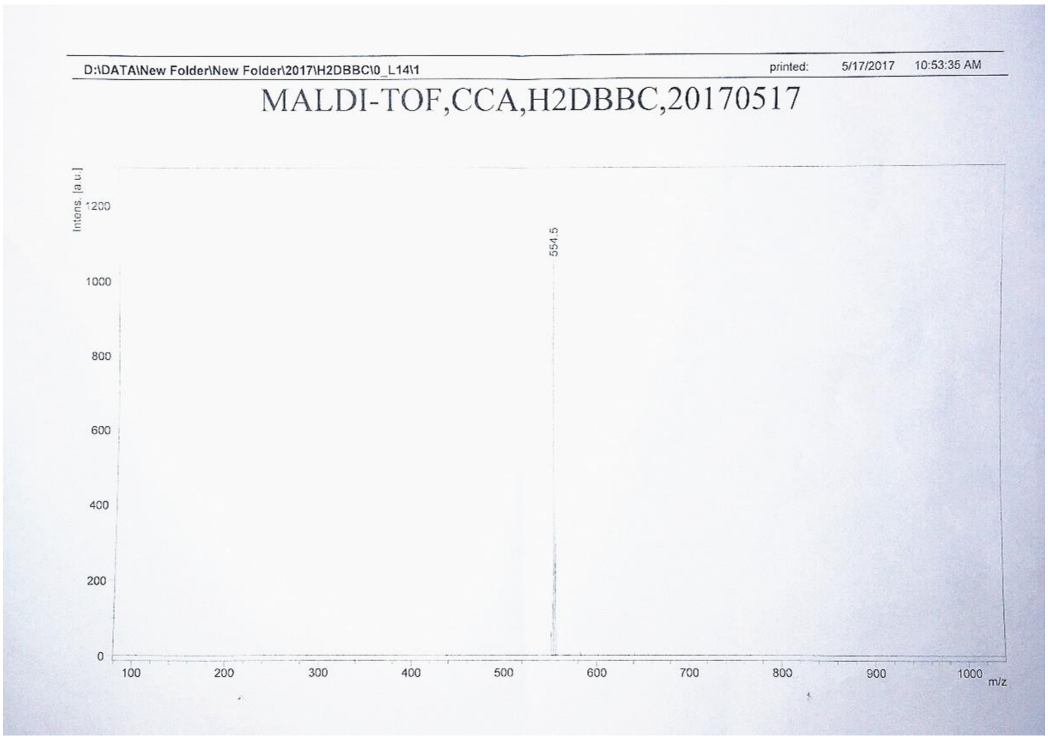

Figure S9. MALDI-TOF MS of H_2DBBC.

Synthesis and characterization of the DBBC-UiO NMOF

To a solution of H_2DBBC (3.5 mg,0.006mmol) in 1ml N, N-dimethylformamide (DMF), a solution of $HfCl_4$ (2mg, 0.006mmol) in 1ml DMF and 0.15 mL of acetic acid (2.6mmol) was added. The reaction mixture was then sealed in a glass vial and heated in an oven at 90° C for three days. After cooling to room temperature, a precipitate was obtained by centrifugation, which was then washed with DMF, triethylamine/ethanol (1:20 V/V) and ethanol sequentially to obtain the desired crystals.

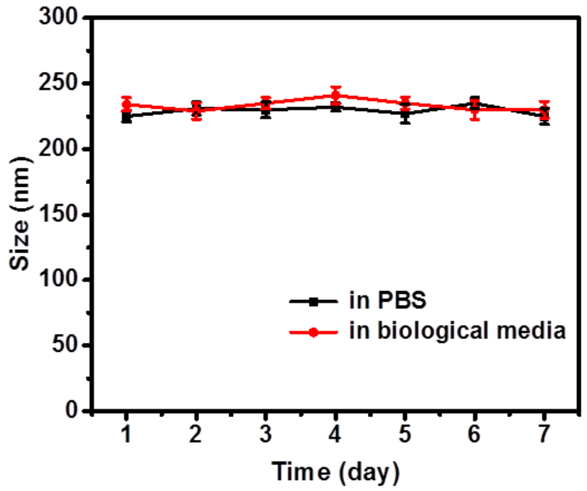

Figure S10. Hydrodynamic size distribution of DBBC-UiO MOF in PBS (10 mM, pH 7.4) and biological media foe 7 consecutive days.

Formula	$Zr_6(O)_4(OH)_4(Zn\text{-}DPDBP)_6$	Absorption coeff (mm^{-1})	0.223
Fw	5263.82	F(000)	10640.0
Temperature (K)	293	θ Range data collection	1.01-13.00
Wavelength (Å)	0.41328	Limiting indices	-39<=h<=41
Crystal system	cubic		-42<=k<=32
Space group	$Fm\overline{3}m$		-38<=I<=41
a, Å	38.758(2)	Reflection collected	81986
b, Å	38.758(2)	Independent reflections	1987
c, Å	38.758(2)	R(int)	0.0936
α °	90	Data/restraints/parameters	1987/35/59
β °	90	Goodness-of-fit on F^2	2.324
γ °	90	Final R indices [I>2σ(I)]	R1 = 0.0896
V, $Å^3$	58224(5)		wR2=0.2592
Z	4	R indices (all data)	R1 = 0.0940
Density (calcd. g/cm^3)	0.600		wR2=0.2644

Table S1. Crystal structure information of $Zr_6O_4(OH)_4(Zn\text{-}DPDBP)_6$.

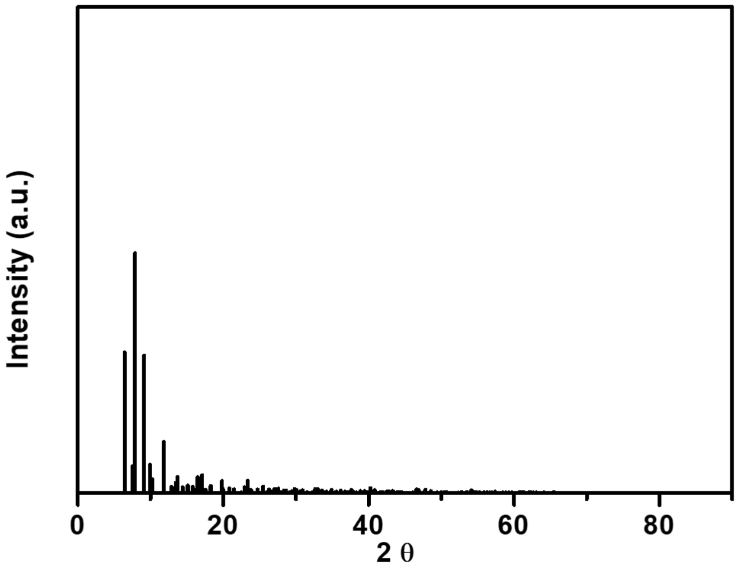

Figure S11. PXRD pattern of $Zr_6O_4(OH)_4(Zn\text{-}DPDBP)_6$.

DBBC-UIO adopts the same structure of $Zr_6O_4(OH)_4(Zn\text{-}DPDBP)_6$ from the PXRD analysis (Figure S11). Meanwhile, the ligand length of DPDBP in the $Zr_6O_4(OH)_4(Zn\text{-}DPDBP)_6$ is similar to the H_2DBBC in the proposed DBBC-UiO.

Thus, we speculated the single structural unit of DBBC-UiO using Crystal structure software Materials Studio.

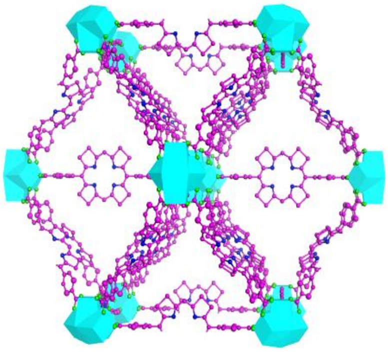

Figure S12. A single structural unit of DBBC- UiO. The which viewed from the [110] direction (light blue polyhedron: Hf^{4+}, purple: C, dark blue: N, green: O and hydrogen is omitted).

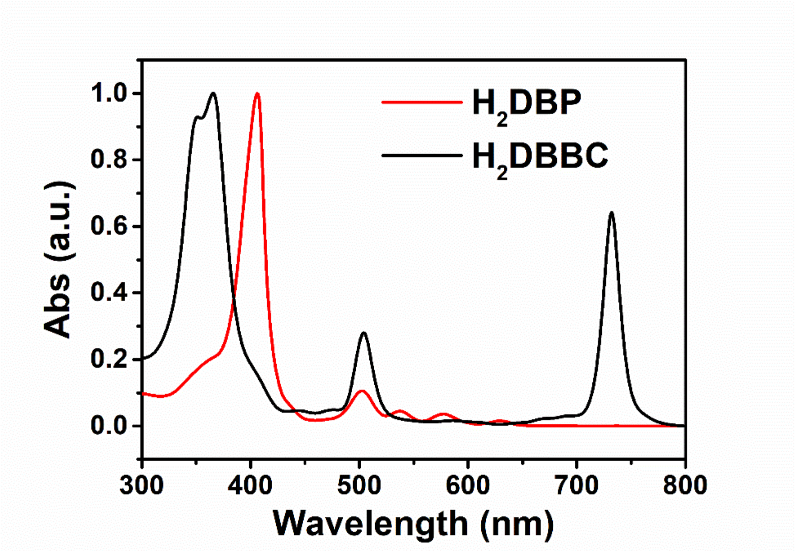

Figure S13. The normalized UV−vis absorbance of H_2DBP and H_2DBBC.

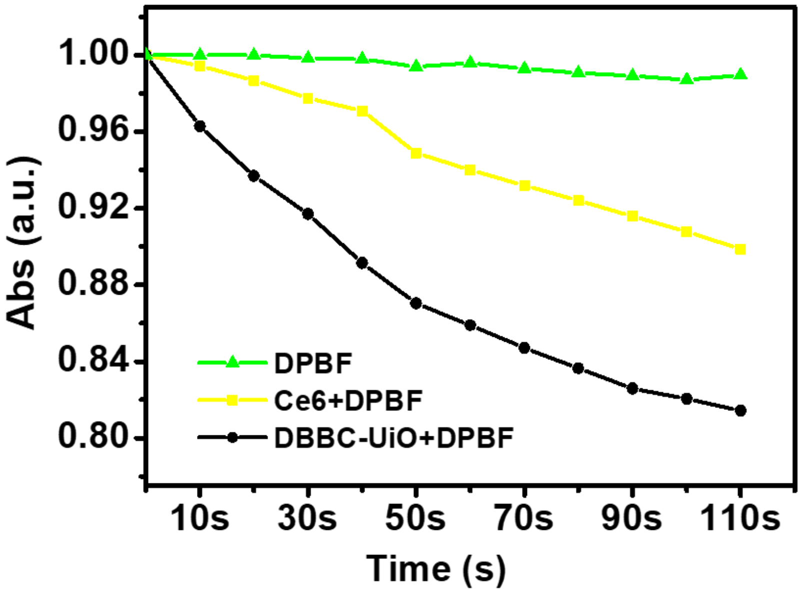

Figure S14. Relative absorbance of DPBF at 425 nm in PBS, Ce-6 and DBBC-UiO exposed for110s under NIR laser.

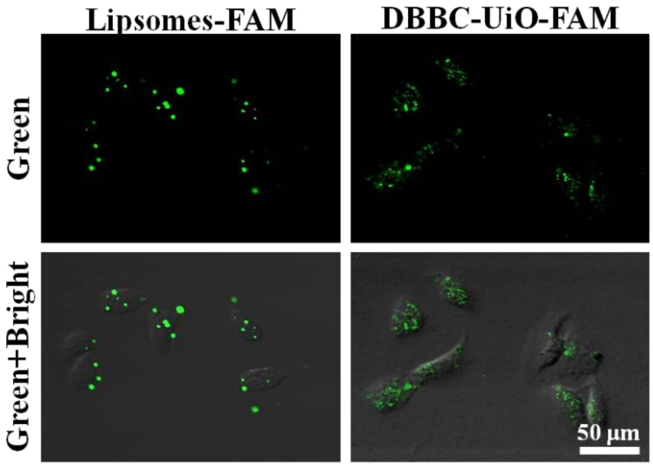

Figure S15. The cellular uptake of resultant DBBC-UiO MOF nanosheet and commercial Lipofectamine®2000 in MCF-7 cells. The DBBC-UiO and the commercial Lipofectamine®2000 were labeled by FAM (green).

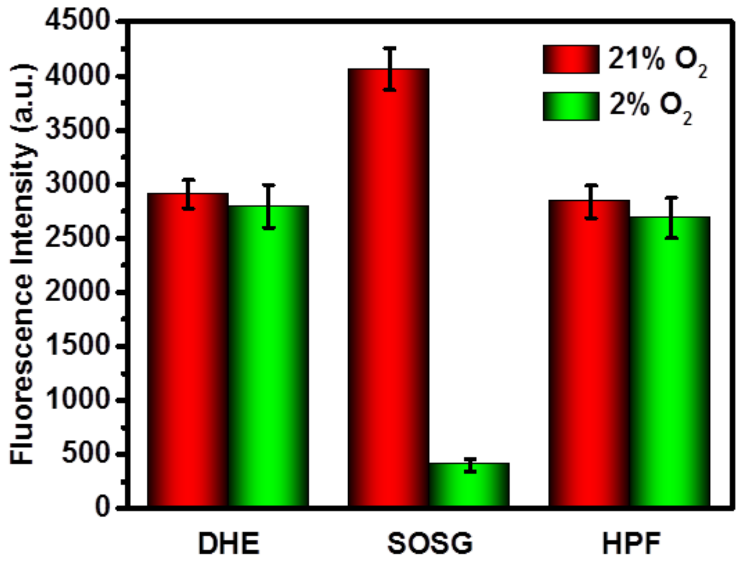

Figure S16. The corresponding fluorescence intensities of ROS in MCF-7 cells exposed on NIR laser in normoxia and hypoxia environments.

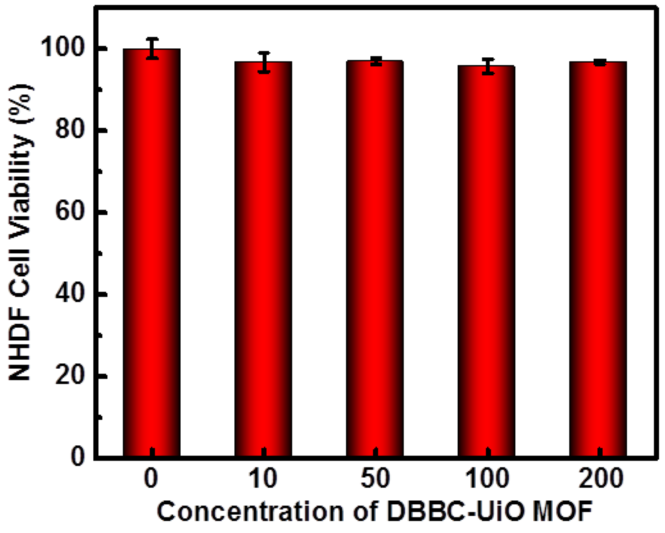

Figure S17. The cytotoxicity of NHDF cells incubated with different concentration of DBBC-UiO MOF.

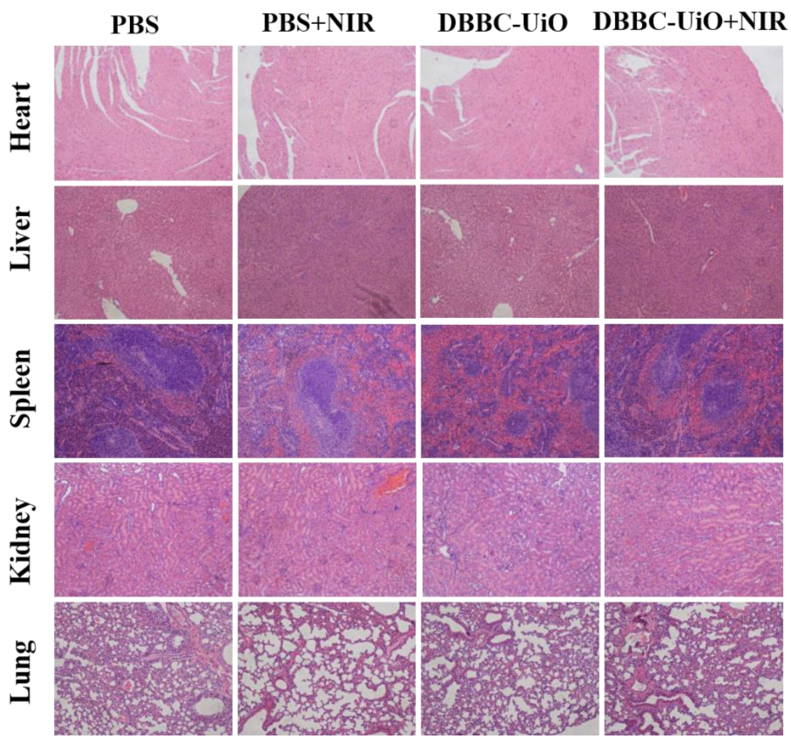

Figure S18. Tissue slices of major organ, that taken from the aforementioned photodynamically treated mice, stained by (H&E).
